# Cerebrovascular Disease-Related Mortality Trends Among Adults in the United States: A Retrospective Population-Based Study

**DOI:** 10.3390/jcm15135204

**Published:** 2026-07-03

**Authors:** Mason Klisares, John Osborne, Kyle Gilkeson, Ameya Chinawalkar, Maddison Weber, Mohamed Azouz, Lauren Hastings, William Thomson, Ali Bin Abdul Jabbar, Ali Al-Salahat, Ahmed Aboeata

**Affiliations:** 1 Department of Medicine, Division of Internal Medicine, Creighton University School of Medicine, Omaha, NE 68124, USA; 2 Department of Neurology, Creighton University School of Medicine, Omaha, NE 68124, USA; 3 Department of Medicine, Division of Cardiovascular Disease, Creighton University School of Medicine, Omaha, NE 68124, USA

**Keywords:** stroke, cerebrovascular disease, trends, disparities, adults, mortality

## Abstract

**Background/Objectives**: Cerebrovascular disease (CVD) is the fifth leading cause of death in the U.S. and poses marked health ramifications. Improvements in CVD-related mortality rates have been observed since the 1970s, but incidence remains high. It is imperative to analyze demographic disparities and trends in CVD-related mortality to inform better efforts to reduce the burden of CVD. This study aimed to examine demographic disparities in CVD-related mortality across age groups, biological sex, race/ethnicity, and regions from 1999 to 2024. **Methods**: This was a retrospective population-based study using the Centers for Disease Control and Prevention Wide-Ranging Online Data for Epidemiologic Research (CDC WONDER). The CDC WONDER was used to extract crude and age-adjusted mortality rates (AAMRs) for CVD-related deaths in the US. Adults aged 25 years and older were included in the study. Joinpoint analysis software was used to calculate the annual percent change (APC) and the average annual percent change (AAPC) for mortality trends. **Results**: There was a total of 6,541,598 CVD-related deaths from 1999 to 2024 in adults aged 25 years and older. The trend in overall CVD-AAMR steadily declined until 2014, then gradually increased, accelerated, and eventually peaked in 2021 during the COVID-19 pandemic. Male individuals experienced a higher mortality rate and comprised 43% of crude deaths vs. 57% of female individuals across the study period. When stratified by race/ethnicity, non-Hispanic (NH) Black individuals had an AAMR that was nearly 1.5 times that of the next-highest group at 148.37 (146.89 to 149.85) per 100,000 people in 2024. The South had the highest AAMR and experienced 39.4% of deaths out of the four regions. All demographics experienced an increase in mortality during the pandemic, but the Northeast region experienced an earlier peak in 2020 as opposed to all other groups, which saw a peak in 2021. **Conclusions**: CVD-related mortality declined significantly in adults from 1999 to 2024. Differences across demographic and regional subgroups have narrowed; however, significant disparities persist. These disparities will require comprehensive, concerted efforts to improve cerebrovascular health, outcomes, and equity among the young and middle-aged populations in the US.

## 1. Introduction

Cerebrovascular disease (CVD), which comprises stroke, transient ischemic attack (TIA), and other cerebrovascular disorders affecting the brain, poses a public health concern. With approximately 795,000 new or recurrent strokes yearly, it is the fifth leading cause of death in the U.S. [1]. CVD-related mortality has steadily declined since the 1970s due to improvements in primary and secondary prevention and advances in acute and post-acute care [2,3]. However, pre-COVID-19 data indicate that the decline in CVD mortality reached an inflection point and has reversed since the early 2010s. This may be due to an increasing incidence among those under the age of 75 years and the incidence of hemorrhagic stroke [4,5]. Differences in sex, race/ethnicity, and geographic location may exacerbate the observed age-related disparities. Trends in the most recent CVD mortality rates (including the year 2024) in adults aged 25 and greater have not been studied. We used the Centers for Disease Control and Prevention Wide-Ranging Online Data for Epidemiologic Research (CDC WONDER) database to examine trends in CVD-related mortality among adults greater than age 25 in the U.S. from 1999 to 2024. We also stratified trends by demographic and regional subgroups to identify disparities by biological sex, race/ethnicity, and region.

## 2. Methods

### 2.1. Data Source

The Centers for Disease Control and Prevention Wide-Ranging Online Data for Epidemiologic Research (CDC WONDER), a public database, was used to extract data for this study [6]. The multiple causes of death data files allowed for the extraction of death certificates with CVD listed as either the underlying or contributing cause of death in adults, aged 25 years and older. Population data were obtained for the years 1999–2024. The International Classification of Diseases 10th Revision (ICD-10) codes, I60–I69, which include subarachnoid hemorrhage (I60), intracerebral hemorrhage (I61), other nontraumatic intracranial hemorrhage (I62), cerebral infarction (I63), stroke not specified as hemorrhage or infarction (I64), occlusion and stenosis of precerebral and cerebral arteries (I65–I66), other cerebrovascular diseases (I67), other cerebrovascular disorders (I68), and sequelae of cerebrovascular disease (I69), were used to identify CVD. Therefore, the present study represents overall cerebrovascular disease-related mortality rather than mortality from individual stroke subtypes.

### 2.2. Populations and Subgroups

The targeted population includes those who passed away from CVD as either an underlying or contributing cause in those aged 25 years and older from 1999 to 2024. Data were stratified by age group, biological sex, race/ethnicity, census region, and state. Age groups were assessed as 10-year cohorts (25–34, 35–44, 45–54, 55–64, 65–74, 75–84, and 85+). Biological sex was divided into male and female. Race/ethnicity was divided into Non-Hispanic (NH) White, NH Black, Non-Hispanic Asian or Pacific Islander (NH-API), Non-Hispanic American Indian (NH-AI) or Alaska Native (NH-AN), or Hispanic. Census regions classified by the U.S. Census Bureau include the Midwest, West, Northeast, and South [7]. States include all 50 U.S. states and the District of Columbia.

### 2.3. Statistical Analysis

To understand mortality trends, the age-adjusted mortality rate (AAMR) and crude mortality rate were calculated per 100,000 people and reported annually from 1999 to 2024. The U.S. 2000 standard population, a widely recognized standard population, was used to calculate the AAMR [8]. The specific subgroups used to examine mortality trends were sex, race, census region, and state whereas crude deaths and age groups were analyzed using the crude mortality rate. Joinpoint trend analysis software (version 5.4.0; National Cancer Institute, Bethesda, MD, USA) was used to fit best-fit lines and calculate the annual percentage change (APC) [9]. The average annual percentage change (AAPC) with 95% confidence intervals (CIs) was then calculated for the entire study period. The software calculated the statistical significance of the APCs and AAPCs with 95% CIs using the Monte Carlo Permutation method. A two-tailed T-test with *p* < 0.05 was used to determine whether a significant change in mortality (i.e., greater than or less than 0) was observed for each time interval.

## 3. Results

**Figure 1:**
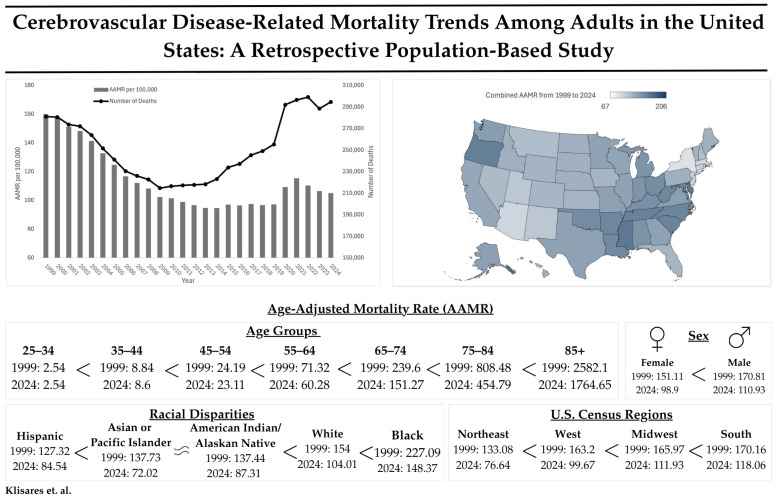
Central Illustration. Summary of the key findings for stroke-related mortality in the US from 1999 to 2024. AAMR: age-adjusted mortality rate.

**Figure 2:**
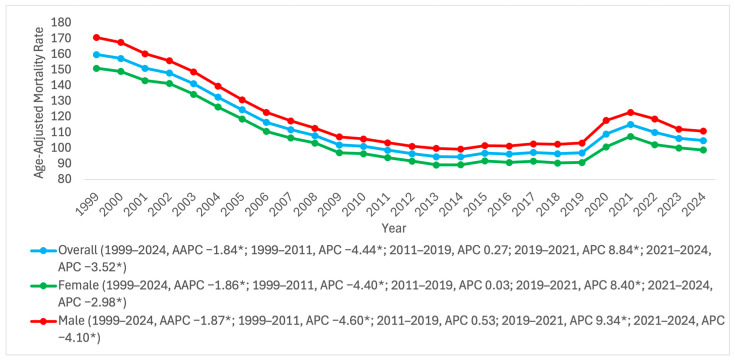
Stroke-related mortality rates in the United States, 1999 to 2024, stratified by overall and sex. AAMR: age-adjusted mortality rate; APC: Annual percentage change; AAPC: average annual percentage change; * = significantly different from 0 with *p* < 0.05.

**Figure 3:**
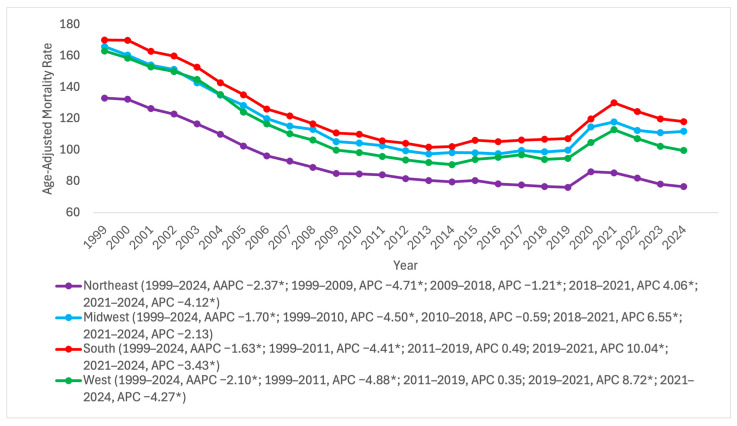
Stroke-related mortality rates in the United States, 1999 to 2024, stratified by US Census Regions. AAMR: age-adjusted mortality rate; APC: Annual percentage change; AAPC: average annual percentage change; * = significantly different from 0 with *p* < 0.05.

**Figure 4:**
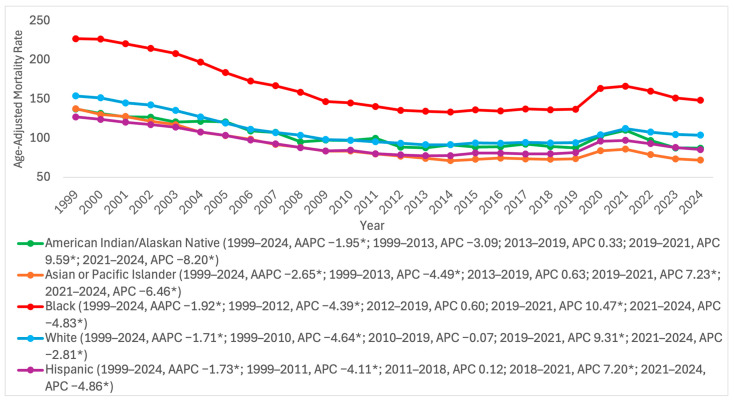
Stroke-related mortality rates in the United States, 1999 to 2024, stratified by race and ethnicity. AAMR: age-adjusted mortality rate; APC: Annual percentage change; AAPC: average annual percentage change; NH: non-Hispanic; * = significantly different from 0 with *p* < 0.05.

**Figure 5:**
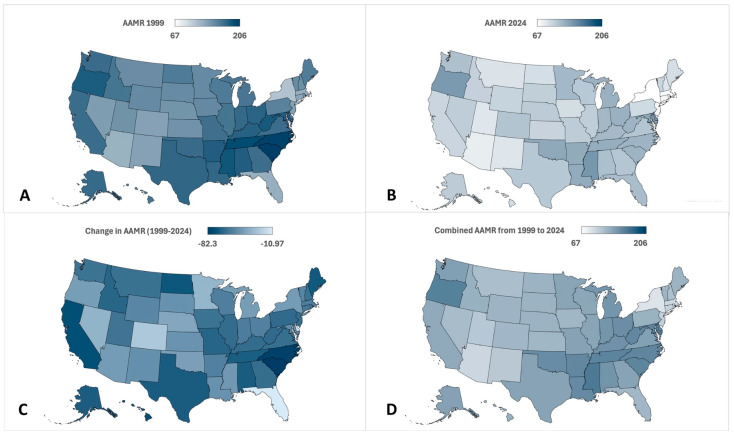
State-level change in Stroke-related mortality rates in the United States, 1999 to 2024, (**A**) AAMR in 1999, (**B**) AAMR in 2024, (**C**) Change in AAMR from 1999 to 2024, (**D**) AAMR from 1999 to 2024. AAMR: age-adjusted mortality rate.

Summarized in Figure 1, Figure 2, Figure 3, Figure 4 and Figure 5 and Table 1. See the Supplemental Files for further details.

### 3.1. Overall and Crude Deaths

There were a total of 6,541,598 CVD-related deaths from 1999 to 2024 in adults aged 25 years and older. There were 281,040 total deaths in 1999, which decreased annually until reaching a nadir in 2009 of 214,716 deaths [Figure 1 and Figure 2]. Annual deaths would stabilize over the next 3 years, until 2014, when they would increase, peaking during the COVID-19 pandemic in 2022 at 299,119. From 1999 to 2019, the AAMR per 100,000 decreased by 39%, from 159.86 (95% CI 159.27 to 160.45) to 97.06 (95% CI 96.68 to 97.44). During the pandemic (2019 to 2021), AAMR increased by 19%, from 97.06 (95% CI 96.68 to 97.44) to 115.2 (95% CI 114.78 to 115.62). Following the peak crude death rate in 2022, death rates would decrease in 2023 and then increase by a similar margin in 2024. The AAPC from 1999 to 2024 was –1.84 * (95% CI, −1.69 to −2.04) [Figure 2].

### 3.2. Disparities in Biological Sex

Females experienced 57% of all crude deaths during the study period, at 3,727,844 deaths, compared to males who experienced 43% at 2,813,754 deaths. During this period, males experienced a consistently higher AAMR, and both sexes showed similar rates of mortality decline [Figure 2]. Male AAMR from 1999 to 2024 decreased by 35% from 170.81 (95% CI 169.79 to 171.82) to 110.93 (95% CI 110.33 to 111.53), and female AAMR decreased by 35% from 151.11 (95% CI 150.39 to 151.84) to 98.9 (95% CI 98.41 to 99.39) [Table 1]. During the pandemic, both sexes experienced similar increases in AAMR, with males increasing by 19% and females increasing by 18%. Following the pandemic period from 2021 to 2024, males experienced a decrease of 10%, from 122.93 (95% CI 122.27 to 123.6) to 110.93 (95% CI 110.33 to 111.53), and females experienced a decrease of 8%, from 107.58 (95% CI 107.05 to 108.12) to 98.9 (95% CI 98.41 to 99.39).

### 3.3. Race and Ethnicity-Related Disparities

NH Black or African American individuals had a disproportionately higher AAMR compared to all other races across the study period [Figure 4]. In 1999, NH Black AAMR was 227.09 (95% CI 224.61 to 229.57), around 47% higher than that of NH White individuals, who had the second-highest AAMR at that time, 154 (95% CI 153.37 to 154.62). Throughout the study period, NH Black individuals had an AAPC of −1.92 (−1.04 to −2.80), and AAMR would decline until 2009, where deaths would stabilize for 3 years until seeing a gradual increase, followed by a sharper increase during the pandemic. Overall, NH Black individuals would see a 35% decrease in AAMR from 1999 to 2024, from 227.09 (95% CI 224.61 to 229.57) to 148.37 (95% CI 146.89 to 149.85). Among NH American Indian/Alaska Native individuals, the AAMR decreased by 36%, from 137.44 (95% CI 128.02 to 146.87) to 87.31 (95% CI 82.97 to 91.85). Hispanic groups saw a 33% decrease in AAMR, from 127.32 (95% CI 124.74 to 129.89) in 1999 to 85.46 (95% CI 84.37 to 86.57) in 2024. AAMR in NH White individuals decreased by 32%, from 154 (95% CI 153.37 to 154.62) in 1999 to 104.01 (95% CI 103.56 to 104.46) in 2024. NH Asian and Pacific Islanders’ AAMR decreased by 48%, from 137.73 (95% CI 133.75 to 141.71) in 1999 to 72.02 (95% CI 70.7 to 73.36) in 2024. The pandemic worsened disparities across all groups, with NH American Indian and Alaskan Natives seeing the largest increase in AAMR from 2019 to 2021 (26%), followed by NH Black individuals with an increase in AAMR of 21%.

### 3.4. Census Region

U.S. census regions showed similar trends throughout the study period, with the South having the highest AAMR. From 1999 to 2024, there were 2,578,582 CVD-related deaths in the South, which accounted for 39.4% of all deaths within the study period. The region with the second highest deaths was the Midwest, with 1,572,873 deaths, accounting for 24% of all deaths. This was followed by the West (20.9%) and the Northeast (16.2%). Although the Northeast experienced the lowest AAMR throughout the study period, it would see the largest improvement in AAMR with an AAPC of −2.37 (−2.23 to −2.55) [Figure 3]. All regions experienced a decrease in AAMR from 1999 to 2019, followed by an increase from 2019 to 2021. The South experienced the largest increase in AAMR during this period, at 21%, from 107.29 (95% CI: 106.63 to 107.95) to 130.04 (95% CI: 129.31 to 130.77). From 2021 to 2024, all regions saw a decrease in AAMR.

### 3.5. State-Level Differences

State-level disparities followed regional patterns: the highest AAMRs between 1999 and 2024 were in the South, whereas the lowest values were in the Northeast. States with the highest combined AAMR from 1999 to 2024 included Mississippi (164.15), Oregon (158.72), Tennessee (156.01), South Carolina (155.75), and Maryland (155.27) [Figure 5, Supplement Table S6]. States with the lowest AAMR were Rhode Island (101.24), Connecticut (100.68), Arizona (96.79), Massachusetts (96.27), and New York (87.75). All U.S. states saw a decrease in AAMR from 1999 to 2024, with South Carolina (−82.3), North Carolina (−80.51), Hawaii (−75.51), California (−74.49), and North Dakota (−71.53) experiencing the most significant declines [Figure 5, Supplement Table S6].

## 4. Discussion

### 4.1. Overall

This study highlights an increase and identifies substantial disparities in CVD-related mortality among adults in the U.S. from 1999 to 2024. Overall, CVD-AAMR declined significantly from 1999 to 2014, then gradually increased from 2014 to 2019. This trend accelerated during the COVID-19 pandemic, peaked in 2021, and has since been gradually declining. Male individuals consistently had a higher mortality throughout the study period and experienced a greater absolute increase in AAMR during the pandemic compared to women. Across racial/ethnic subgroups from 1999 to 2019, the NH Black population saw the largest decline in AAMR, while the NH AI/AN population saw the smallest decline. During the pandemic, NH Black and NH AI/AN subgroups also experienced the largest increases in AAMR. Regionally, the South consistently had the highest AAMR across the study period and had the largest absolute increase in AAMR from 2019 to 2021. The West experienced the largest decline in AAMR throughout the study period. The Midwest was the only region with an increase in AAMR in 2024. Adults aged 85 years and older consistently had much higher mortality rates, the largest increase in overall mortality during the COVID-19 pandemic, and a much larger improvement in mortality throughout the study period.

### 4.2. Increasing CVD Mortality

The decline in CVD mortality observed in this study from 1999 to 2019 continued a decades-long trend dating back to the 1970s [2]. Advances in primary and secondary prevention have been instrumental in improving CVD-related mortality [3]. Public health initiatives aimed at primary prevention and improved management of CVD risk factors, including hypertension, hyperlipidemia, diabetes mellitus, and smoking cessation, have led to declines in CVD incidence and thus mortality [3]. Furthermore, increased adoption of anticoagulation in patients with atrial fibrillation has led to a reduction in incident ischemic stroke, which impacts ischemic stroke mortality rates in the population [3,10]. Advances in acute and post-acute CVD treatment, such as intravenous (IV) thrombolysis, endovascular intervention, and the improvement and continuous refinement of stroke systems of care (e.g., telemedicine, stroke units/teams, and primary/secondary stroke centers) have all significantly contributed to the decline in mortality [3,11]. Despite advancements in the management of CVD, recent years have shown a reversal in the decline of CVD-related mortality. This is partly attributed to an increase in CVD mortality among those aged 35–64 years and a slower rate of mortality decline among those aged 65 years and older. Additionally, traditional CVD risk factors are increasingly prevalent among younger populations [4,5]. This is compounded by the fact that a larger proportion of younger individuals remain undertreated for traditional CVD risk factors, possibly because the absolute CVD risk thresholds used in clinical practice underestimate the risk in those individuals [12]. Specifically, the adequate treatment of hypertension, the most significant modifiable risk factor for CVD, has deteriorated in the United States since 2013, including in younger adults [13]. The inflection point in CVD mortality in 2014 coincides with increased deaths among adults in the U.S. due to drug overdoses. With an increase in illicit drug use and binge alcohol drinking during adolescence and young adulthood, these lifestyle choices may be contributing to the rise in CVD mortality in the past decade [5]. Tobacco use remains a significant modifiable risk factor for stroke, approximately doubling ischemic stroke risk and tripling subarachnoid hemorrhage risk, with a causal relationship supported by Mendelian randomization [14]. Despite a historic decline in U.S. smoking prevalence to 11%, tobacco-associated stroke mortality has risen nearly 10-fold from 1999 to 2023, with the burden concentrated among NH Black individuals, rural populations, the Midwest and Southeast, and older adults. Furthermore, several nontraditional risk factors have emerged as potential contributors to the increased incidence of stroke in younger populations, including exposure to air pollution, rising ambient temperatures, long working hours, decreased levels of physical activity, psychosocial stress, autoimmune disease, depression, and illicit drug use [15]. However, the data surrounding this are purely correlational and warrant further investigation. Cancer diagnosis is associated with a significantly higher risk of CVD, with some studies reporting a 66% higher relative risk compared to cancer-free populations. Pancreatic and lung cancers carry the highest risk of stroke overall [16]. Risk is also higher in certain populations, particularly those younger than 45 years and females [17]. Lastly, trends in CVD subtypes help explain recent mortality trends. While ischemic stroke incidence rose by 13.0%, the incidence of intracerebral hemorrhage (ICH) and subarachnoid hemorrhage (SAH) grew by 39.8% and 50.9%, respectively [4].

### 4.3. Impact of the COVID-19 Pandemic

The rise in CVD-related mortality during the COVID-19 pandemic could be explained by a combination of direct effects of SARS-CoV-2 infection and pandemic-related disruptions in CVD care. Adverse cardiovascular effects of SARS-CoV-2 include activation of inflammatory and thrombotic cascades, direct viral injury to myocytes and endothelium, and worsening of underlying atherosclerotic and structural abnormalities [18]. These adverse effects contribute to increased arterial and venous thrombosis, thereby directly contributing to increased CVD mortality. Existing literature has shown a significant increase in in-hospital mortality in stroke patients infected with SARS-CoV-2 compared to noninfected individuals, especially in cryptogenic ischemic stroke [19,20]. This is due to increased likelihood of mechanical ventilation requirements, venous thromboembolism (VTE), acute myocardial infarction (AMI), cardiac arrest, septic shock, and acute kidney injury (AKI) in patients with COVID-19 compared to non-infected patients [20].

The COVID-19 pandemic had a significant impact on healthcare systems worldwide, including in the U.S. Emergency departments and hospitals were overwhelmed, with people avoiding medical facilities to prevent COVID-19 exposure [20,21]. It has also been proposed that the reduced social contact of the early COVID-19 pandemic led to a lower rate of third-party detection of CVD symptoms that patients would not have noticed on their own [21]. Furthermore, an increase in CVD-related mortality temporarily correlated with a marked reduction in emergency medical services (EMS) calls for stroke symptoms during the early days of the COVID-19 pandemic [21]. This decrease in stroke-related EMS calls led to reduced early detection of stroke-like symptoms, early reperfusion therapies, and monitoring/treatment of poststroke complications, all of which are well known to improve early survival [21]. Lastly, patients avoided clinic appointments more frequently, which may have led to reduced use of preventive care. This likely led to decreased identification and poorer management of CVD risk factors.

### 4.4. Sex Disparities

Male individuals consistently had a higher mortality throughout the study period and had a larger absolute increase in AAMR during the COVID-19 pandemic. Risk factors for CVD are more prevalent among males than among females [22]. CVD incidence is also higher in males at younger age groups, leading to increased mortality. CVD incidence in females exceeds that in males after age 75, possibly due to increased female life expectancy [23]. Furthermore, higher levels of estrogen in premenopausal women are largely responsible for protection against CVD [24]. The greater absolute increase in CVD-related mortality among male individuals during the COVID-19 pandemic was possibly related to higher levels of systemic inflammation, which are hypothesized to increase the likelihood of ischemic stroke [25].

### 4.5. Racial and Ethnic Disparities

CVD-related mortality declined across all racial/ethnic subgroups during the study period, but significant disparities persisted. The greatest absolute decline in mortality was seen in the NH Black subgroup. However, their overall mortality remains markedly higher than that of others. This can be partly attributed to higher prevalence and poorer control of CVD risk factors among NH Black individuals [26]. Social determinants of health also contribute to these disparities. As seen in The Reasons for Geographic and Racial Differences in Stroke (REGARDS) Study, NH Black people had higher levels of psychosocial stress, which is a risk factor for cardiovascular disease. This was likely due to stress-related features of the neighborhood’s physical environment, neighborhood safety, perceived stress, discrimination, and lower levels of neighborhood social cohesion [27]. Dietary intake and consumption of ultra-processed foods, which commonly contain food additive emulsifiers, have also been associated with increased cerebrovascular disease risk. This association appears stronger among Black individuals than White individuals [28]. Cognitive impairment and vascular dementia are also important long-term sequelae of cerebrovascular disease. Among dementia subtypes, vascular dementia is associated with the shortest median survival following diagnosis. Older age at symptom onset and male sex are associated with higher mortality, while vascular dementia is more prevalent among Black individuals than White individuals in the United States, highlighting demographic disparities that parallel those observed in our study [29,30].

The smallest absolute decline was observed in the NH AI/AN subgroup, which experienced a notable increase in CVD-related mortality from the 2013/2019 nadir to 2023, followed by a new nadir in 2024. Since 2000, the prevalence of most cardiovascular risk factors has increased in Native Americans. Among those, the increases in hypertension, hyperlipidemia, and tobacco use were statistically significant [27]. By 2016, Native Americans had the highest prevalence of diabetes, coronary artery disease, and tobacco and alcohol use among all ethnic subgroups [27]. Additionally, approximately one-third of the NH AI/AN population receives health care from the Indian Health Service. Many of their facilities are small, underfunded, and lack sufficient resources to provide optimal care in emergencies, such as advanced therapies for timely reperfusion in the setting of acute CVD. They are also located in rural areas and often require patients to travel long distances, which is costly in acute CVD care [31].

### 4.6. Regional Disparities

Regionally, the South persistently had the highest AAMR throughout the study period. The Southern United States encompasses the “Stroke Belt,” a region known to have the highest incidence of CVD in the U.S. [32]. Of the classic CVD risk factors, hypertension and diabetes mellitus are most prevalent in the South. However, smoking is most prevalent in the Midwest [32]. The higher CVD-related mortality in the South is also likely attributable to factors beyond the higher prevalence of CVD risk factors. Prior data from the REGARDS study indicate that socioeconomic status, rurality, proximity to stroke centers, and inflammation/infection also may contribute to higher stroke incidence and mortality in the South [4]. Interestingly, race is a potential confounder in the regional mortality trends observed in our study. In 2017, NH Black people comprised ~26% of residents in the Stroke Belt [32]. With this racial subgroup having the highest CVD-related mortality compared to others, as evidenced above, it is clear that race is a contributor to mortality in the South.

The Northeast is the only region in which AAMR peaked in 2020 rather than 2021. This can be partly explained by the greater density of urban areas in the Northeast and the greater intensity of COVID-19 outbreaks experienced in those cities [33]. Additionally, COVID vaccination has been shown to decrease the risk of severe cardiovascular outcomes, with a lower risk of myocardial infarction and stroke after SARS-CoV-2 infection [34]. Higher and earlier vaccination rates in 2021 likely reduced severe cardiovascular outcomes, resulting in a smaller, earlier peak in CVD mortality [35].

The Midwest is the only region that did not see a continued decline in AAMR in 2024. This may be partly explained by a higher prevalence of risk factors such as obesity, cardiovascular-kidney-metabolic (CKM) syndrome, and cigarette smoking in the Midwest [32,36,37]. Rurality, low levels of insurance coverage, high levels of substance abuse, and suicide mortality are social determinants of health associated with higher levels of stroke mortality, especially in younger adults [5]. These are disproportionately higher in the Midwest and South, offering a potential explanation for the increase in mortality seen in the Midwest in 2024.

AAMR was significantly higher among adults aged 85 years and older than in younger age groups. Age is the most powerful non-modifiable risk factor for CVD, with CVD rates doubling each decade after the age of 50 [4,38]. As individuals age, their cumulative exposure to vascular risk factors increases. Additionally, older individuals are more likely to have multiple comorbidities, greater stroke severity, and lower physiologic reserve, all of which contribute to higher stroke mortality [39].

### 4.7. Future Directions

Future research should take a detailed look at long-term stroke mortality trends for each subtype of stroke (e.g., ischemic, hemorrhagic, intraparenchymal hemorrhage, subarachnoid hemorrhage). Mortality and long-term outcomes should also be studied in those who receive acute care, such as IV thrombolysis or mechanical thrombectomy. Future studies may examine differences in trends between CVD risk factors stratified by the various demographic subgroups analyzed in this study.

### 4.8. Strengths and Limitations

This study comprehensively analyzed CVD-related mortality and its disparities from 1999 to 2024. The findings are relevant to public health strategy and CVD prevention guidelines. However, limitations remain in our study. Data obtained from CDC WONDER is derived from death certificates, which use ICD codes to classify diseases; this can lead to misclassification bias and discrepancies in cause-of-death coding across different states and regions. Our study cannot distinguish incident stroke from recurrent stroke or late sequelae. Comorbidities were not accounted for, thus introducing potential confounding. Stroke severity was not accounted for, which limits causal inference. Furthermore, given the nature of the data, establishing a causal link between CVD and COVID-19 infection for the fatalities during the pandemic or any CVD risk factors throughout the study period is not feasible. Socioeconomic factors such as access to healthcare, education level, and income are not captured in the CDC WONDER database, and these factors could significantly influence mortality outcomes. Geographical differences in death certificate reporting and coding practices may lead to inconsistencies, thereby affecting our regional analysis. Our study is subject to the potential for ecological fallacy, and associations observed across geographic regions and race/ethnicity should not be interpreted as reflecting individual-level risk or causal relationships. Lastly, our study used the complete ICD-10 I60–I69 code range, which encompasses ischemic stroke, hemorrhagic stroke, other cerebrovascular disorders, and sequelae of prior cerebrovascular disease. Consequently, our findings reflect overall cerebrovascular disease-related mortality rather than subtype-specific mortality. In particular, inclusion of I69 codes captures deaths related to the long-term sequelae of prior cerebrovascular events rather than acute stroke alone, which may influence interpretation of temporal mortality trends. Future studies evaluating ischemic stroke, hemorrhagic stroke, and cerebrovascular sequelae separately may provide additional clinical insight into subtype-specific mortality patterns.

## 5. Conclusions

CVD-related mortality declined significantly for adults from 1999 to 2014 then gradually increased from 2014 to 2019. This trend accelerated during the COVID-19 pandemic, peaked in 2021, and has gradually declined since then. Although differences in CVD-related mortality across demographic groups narrowed, significant disparities persist. These disparities will require comprehensive efforts to improve CVD outcomes and health equity in the US.

## Figures and Tables

**Table 1:** Demographic Characteristics of Deaths due to Cerebrovascular Disease.

	AAMR 1999 (95% CI)	AAMR 2019 (95% CI)	AAMR 2021 (95% CI)	AAMR 2024 (95% CI)	AAPC from 1999 to 2024 (95% CI)	Percentage Change in AAMR from 1999 to 2024	Percentage Change in AAMR from 1999 to 2019	Percentage Change in AAMR from 2019 to 2021	Percentage Change in AAMR from 2021 to 2024
Overall	159.86 (159.27–160.45)	97.06 (96.68–97.44)	115.2 (114.78–115.62)	104.95 (104.57–105.33)	−1.84 * (−1.69 to −2.04)	−34%	−39%	19%	−9%
Sex stratified									
Male	170.81 (169.79–171.82)	103.38 (102.78–103.99)	122.93 (122.27–123.6)	110.93 (110.33–111.53)	−1.87 * (−1.72 to −2.06)	−35%	−39%	19%	−10%
Female	151.11 (150.39–151.84)	90.96 (90.47–91.44)	107.58 (107.05–108.12)	98.9 (98.41–99.39)	−1.86 * (−1.71 to −2.08)	−35%	−40%	18%	−8%
Race/Ethnicity Stratified									
NH American Indian or Alaska Native	137.44 (128.02–146.87)	87.77 (83.01–92.53)	110.17 (104.81–115.74)	87.31 (82.97–91.85)	−1.95 * (−1.66 to −2.28)	−36%	−36%	26%	−21%
NH Asian or Pacific Islander	137.73 (133.75–141.71)	73.84 (72.34–75.34)	86 (84.41–87.62)	72.02 (70.7–73.36)	−2.65 * (−2.43 to −2.90)	−48%	−46%	16%	−16%
NH Black or African American	227.09 (224.61–229.57)	136.98 (135.5–138.47)	166.4 (164.76–168.06)	148.37 (146.89–149.85)	−1.92 * (−1.04 to −2.80)	−35%	−40%	21%	−11%
NH White	154 (153.37–154.62)	94.39 (93.96–94.82)	112.38 (111.89–112.86)	104.01 (103.56–104.46)	−1.71 * (−0.95 to −2.47)	−32%	−39%	10%	−7%
Hispanic	127.32 (124.74–129.89)	81.75 (80.56–82.93)	97.26 (96–98.53)	85.46 (84.37–86.57)	−1.73 * (−1.54 to −1.93)	−33%	−36%	19%	−12%
Census Region									
Northeast	133.08 (131.91–134.25)	76.2 (75.43–76.98)	85.45 (84.62–86.28)	76.64 (75.89–77.41)	−2.37 * (−2.23 to −2.55)	−42%	−43%	12%	−10%
Midwest	165.97 (164.75–167.2)	99.85 (99.02–100.68)	117.89 (116.98–118.82)	111.93 (111.07–112.80)	−1.70 * (−1.52 to −1.99)	−33%	−40%	18%	−5%
South	170.16 (169.12–171.19)	107.29 (106.63–107.95)	130.04 (129.31–130.77)	118.06 (117.4–118.72)	−1.63 * (−1.48 to −1.81)	−31%	−37%	21%	−9%
West	163.2 (161.85–164.55)	94.66 (93.87–95.46)	112.9 (112.03–113.79)	99.67 (98.89–100.46)	−2.10 * (−1.91 to −2.35)	−39%	−42%	19%	−12%

* Indicates the AAPC is significantly different from 0.

## Data Availability

The data in this study are publicly available in the CDC-WONDER database at https://wonder.cdc.gov.

## Supplementary Materials

The following supporting information can be downloaded at https://www.mdpi.com/article/10.3390/jcm15135204/s1, Table S1. Cerebrovascular Disease-Related Crude Number of Deaths in the US Population Stratified by Sex, Race, Region, Age Group, Census Region, and Urbanization Status in the United States. Table S2. Cerebrovascular Disease-Related Mortality Rates Stratified by Sex, Race, and Age in the United States. Table S3. Cerebrovascular Disease-Related Mortality Rates Stratified by Race. Table S4. Cerebrovascular Disease-Related Mortality Rates Stratified by Age. Table S5. Cerebrovascular Disease-Related Mortality Rates Stratified by Region. Table S6. Cerebrovascular Disease-Related Mortality Rates Stratified by State.

## Abbreviations

The following abbreviations are used in this manuscript:

CVD	Cerebrovascular Disease
TIA	Transient Ischemic Attack
ICH	Intracerebral Hemorrhage
SAH	Subarachnoid Hemorrhage
AMI	Acute Myocardial Infarction
AKI	Acute Kidney Injury
IV	Intravenous
CKM	Cardiovascular-kidney-metabolic
AAMR	Age-Adjusted Mortality Rate
APC	Annual Percentage Change
AAPC	Average Annual Percentage Change
VTE	Venous Thromboembolism
CI	Confidence Interval
NH	Non-Hispanic
NH-AI	Non-Hispanic American Indian
NH-AN	Non-Hispanic Alaskan Native
NH-API	Non-Hispanic Asian or Pacific Islander
ICD-10	International Classification of Diseases 10th Revision
REGARDS	Reasons for Geographic and Racial Differences in Stroke
CDC WONDER	Centers for Disease Control and Prevention Wide-Ranging Online Data for Epidemiologic Research
