# Supplementary material for: Cerebrovascular Disease-Related Mortality Trends Among Adults in the United States: A Retrospective Population-Based Study
Source: J Clin Med. 2026 Jul 3;15(13):5204. doi: 10.3390/jcm15135204 (PMC13362691; doi:10.3390/jcm15135204)
Supplement: Supplementary file 1 [file jcm-15-05204-s001.zip › jcm-4412564-supplementary.pdf]

Supplemental Tables:

Supplemental Table S1. Cerebrovascular Disease-Related Crude Number of Deaths in the US Population Stratified by Sex, Race, Region, Age Group, Census Region, and Urbanization Status in the United States.

Year	Overall	Female	Male	NH American Indian or Alaska Native	NH Asian or Pacific Islander	NH Black or African American	NH White	Hispanics	Northeast	Midwest	South	West
**1999**	281040	167797	113243	911	5077	33022	231040	10293	50114	70723	104101	56102
**2000**	280552	167600	112952	951	5104	33399	229859	10559	50519	68953	105437	55643
**2001**	273707	163162	110545	935	5445	33092	222674	10891	48875	67100	102631	55101
**2002**	272047	162697	109350	965	5485	32651	221143	11158	48119	66613	102088	55227
**2003**	263841	156775	107066	942	5700	32310	212926	11434	46288	63715	99144	54694
**2004**	251502	148604	102898	980	5491	31210	202005	11419	44042	60844	94450	52166
**2005**	241127	141911	99216	1016	5671	30011	192445	11638	41619	58633	91576	49299
**2006**	230464	134618	95846	922	5752	29023	182782	11612	39601	55742	87556	47565
**2007**	226012	131908	94104	952	5662	28755	178688	11687	38798	54438	86489	46287
**2008**	222663	130095	92568	885	5800	28028	175907	11618	37677	54288	84930	45768
**2009**	214716	124181	90535	951	5779	26803	169053	11727	36504	51192	82674	44346
**2010**	216484	125333	91151	941	6037	27046	169863	12250	36912	51424	83546	44602
**2011**	217243	124954	92289	1047	6244	27115	169955	12585	37232	51657	83219	45135
**2012**	217671	124699	92972	982	6466	27133	169537	13035	36892	50975	84359	45445
**2013**	218274	123621	94653	1030	6689	27691	168776	13634	36900	50837	84671	45866
**2014**	223161	126171	96990	1126	6911	28520	171602	14480	36937	52024	87442	46758
**2015**	233867	132041	101826	1160	7546	29999	178390	15970	37822	52686	93489	49870
**2016**	237169	133056	104113	1201	8113	30618	179972	16707	37202	52966	95157	51844

**2017**	245271	136853	108418	1306	8550	32300	185074	17489	37822	55115	98469	53865
**2018**	249021	137650	111371	1351	8904	32975	187019	18261	37839	55611	101830	53741
**2019**	255245	140195	115050	1385	9490	34126	190451	19382	38044	56993	104821	55387
**2020**	291983	157734	134249	1707	11356	42043	212416	23970	43276	65992	120058	62657
**2021**	296436	159276	137160	1686	11296	41727	215528	24372	41832	64799	125257	64548
**2022**	299119	161805	137314	1570	11218	41353	218625	24405	41973	65053	126939	65154
**2023**	288402	156192	132210	1463	10784	39720	210711	23736	39853	63526	122818	62205
**2024**	294581	158916	135665	1562	11385	40539	214407	24648	40254	65974	125431	62922
**Total**	6541598	3727844	2813754	29927	191955	841209	5060848	398960	1062946	1527873	2578582	1372197

Supplemental Table S2. Cerebrovascular Disease-Related Mortality Rates Stratified by Sex, Race, and Age in the United States.

	Year	Age Adjusted Rate	Age Adjusted Rate Lower 95% Confidence Interval	Age Adjusted Rate Upper 95% Confidence Interval	Age Adjusted Rate Standard Error
**Overall**	1999	159.86	159.27	160.45	0.3
**Overall**	2000	157.44	156.86	158.02	0.3
**Overall**	2001	151.09	150.53	151.66	0.29
**Overall**	2002	148.07	147.51	148.62	0.28
**Overall**	2003	141.22	140.68	141.76	0.28
**Overall**	2004	132.65	132.13	133.17	0.26
**Overall**	2005	124.6	124.1	125.1	0.25
**Overall**	2006	116.59	116.11	117.07	0.24
**Overall**	2007	111.97	111.51	112.44	0.24
**Overall**	2008	108.1	107.65	108.55	0.23
**Overall**	2009	102.14	101.71	102.58	0.22
**Overall**	2010	101.25	100.82	101.68	0.22
**Overall**	2011	98.79	98.37	99.21	0.21
**Overall**	2012	96.54	96.13	96.95	0.21
**Overall**	2013	94.62	94.22	95.02	0.2
**Overall**	2014	94.52	94.12	94.91	0.2
**Overall**	2015	96.88	96.48	97.28	0.2
**Overall**	2016	96.31	95.92	96.71	0.2
**Overall**	2017	97.31	96.92	97.7	0.2
**Overall**	2018	96.56	96.18	96.94	0.2
**Overall**	2019	97.06	96.68	97.44	0.19
**Overall**	2020	109.09	108.69	109.49	0.2
**Overall**	2021	115.2	114.78	115.62	0.21

**Overall**	2022	110.18	109.78	110.58	0.2
**Overall**	2023	106.3	105.91	106.69	0.2
**Overall**	2024	104.95	104.57	105.33	0.2
**Female**	1999	151.11	150.39	151.84	0.37
**Female**	2000	149.12	148.4	149.84	0.37
**Female**	2001	143.27	142.57	143.97	0.36
**Female**	2002	141.43	140.74	142.12	0.35
**Female**	2003	134.46	133.79	135.13	0.34
**Female**	2004	126.34	125.69	126.98	0.33
**Female**	2005	118.77	118.15	119.39	0.32
**Female**	2006	110.81	110.21	111.41	0.3
**Female**	2007	106.63	106.05	107.21	0.3
**Female**	2008	103.4	102.83	103.97	0.29
**Female**	2009	97.13	96.58	97.68	0.28
**Female**	2010	96.53	95.98	97.07	0.28
**Female**	2011	94.03	93.5	94.57	0.27
**Female**	2012	91.79	91.27	92.31	0.26
**Female**	2013	89.37	88.86	89.88	0.26
**Female**	2014	89.47	88.97	89.97	0.26
**Female**	2015	91.87	91.37	92.38	0.26
**Female**	2016	90.98	90.48	91.48	0.25
**Female**	2017	91.77	91.27	92.26	0.25
**Female**	2018	90.6	90.11	91.08	0.25
**Female**	2019	90.96	90.47	91.44	0.25
**Female**	2020	100.93	100.42	101.43	0.26
**Female**	2021	107.58	107.05	108.12	0.27
**Female**	2022	102.33	101.83	102.84	0.26
**Female**	2023	100.24	99.74	100.74	0.26

**Female**	2024	98.9	98.41	99.39	0.25
**Male**	1999	170.81	169.79	171.82	0.52
**Male**	2000	167.65	166.66	168.65	0.51
**Male**	2001	160.34	159.38	161.3	0.49
**Male**	2002	155.9	154.96	156.85	0.48
**Male**	2003	148.8	147.89	149.71	0.46
**Male**	2004	139.72	138.86	140.59	0.44
**Male**	2005	131.01	130.18	131.84	0.42
**Male**	2006	122.96	122.17	123.75	0.4
**Male**	2007	117.51	116.75	118.27	0.39
**Male**	2008	112.81	112.07	113.54	0.38
**Male**	2009	107.26	106.55	107.97	0.36
**Male**	2010	105.97	105.27	106.66	0.36
**Male**	2011	103.51	102.83	104.19	0.35
**Male**	2012	101.24	100.58	101.9	0.34
**Male**	2013	99.91	99.26	100.56	0.33
**Male**	2014	99.47	98.84	100.11	0.32
**Male**	2015	101.69	101.06	102.33	0.32
**Male**	2016	101.45	100.82	102.07	0.32
**Male**	2017	102.81	102.18	103.43	0.32
**Male**	2018	102.6	101.99	103.21	0.31
**Male**	2019	103.38	102.78	103.99	0.31
**Male**	2020	117.8	117.16	118.44	0.33
**Male**	2021	122.93	122.27	123.6	0.34
**Male**	2022	118.73	118.09	119.37	0.33
**Male**	2023	112.22	111.6	112.84	0.31
**Male**	2024	110.93	110.33	111.53	0.31

Supplemental Table S3. Cerebrovascular Disease-Related Mortality Rates Stratified by Race

Race	Year	Age Adjusted Rate	Age Adjusted Rate Lower 95% Confidence Interval	Age Adjusted Rate Upper 95% Confidence Interval	Age Adjusted Rate Standard Error
**American Indian or Alaska Native**	1999	137.44	128.02	146.87	4.81
**American Indian or Alaska Native**	2000	131.56	122.79	140.32	4.47
**American Indian or Alaska Native**	2001	127.37	118.8	135.95	4.38
**American Indian or Alaska Native**	2002	126.89	118.42	135.36	4.32
**American Indian or Alaska Native**	2003	120.81	112.65	128.97	4.16
**American Indian or Alaska Native**	2004	121.7	113.63	129.78	4.12
**American Indian or Alaska Native**	2005	120.94	113.03	128.84	4.03
**American Indian or Alaska Native**	2006	109.32	101.84	116.8	3.82
**American Indian or Alaska Native**	2007	107.43	100.18	114.67	3.69
**American Indian or Alaska Native**	2008	95.32	88.65	101.99	3.4
**American Indian or Alaska Native**	2009	97.63	91.03	104.24	3.37
**American Indian or Alaska Native**	2010	97.2	90.63	103.78	3.35

**American Indian or Alaska Native**	2011	99.93	93.54	106.32	3.26
**American Indian or Alaska Native**	2012	88.73	82.9	94.57	2.98
**American Indian or Alaska Native**	2013	87.77	82.14	93.39	2.87
**American Indian or Alaska Native**	2014	91.66	86.06	97.26	2.86
**American Indian or Alaska Native**	2015	88.69	83.37	94.01	2.71
**American Indian or Alaska Native**	2016	88.97	83.75	94.19	2.66
**American Indian or Alaska Native**	2017	92.46	87.28	97.65	2.65
**American Indian or Alaska Native**	2018	89.43	84.5	94.35	2.51
**American Indian or Alaska Native**	2019	87.77	83.01	92.53	2.43
**American Indian or Alaska Native**	2020	102.64	97.63	107.64	2.55
**American Indian or Alaska Native**	2021	110.17	104.81	115.74	2.77
**American Indian or Alaska Native**	2022	97.07	92.23	102.11	2.5
**American Indian or Alaska Native**	2023	87.57	83.05	92.28	2.33
**American Indian or Alaska Native**	2024	87.31	82.97	91.85	2.25
**Asian or Pacific Islander**	1999	137.73	133.75	141.71	2.03

**Asian or Pacific Islander**	2000	130.46	126.72	134.19	1.91
**Asian or Pacific Islander**	2001	127.7	124.17	131.23	1.8
**Asian or Pacific Islander**	2002	121.77	118.43	125.12	1.71
**Asian or Pacific Islander**	2003	117.51	114.35	120.68	1.62
**Asian or Pacific Islander**	2004	107.82	104.87	110.77	1.51
**Asian or Pacific Islander**	2005	103.46	100.68	106.24	1.42
**Asian or Pacific Islander**	2006	98.63	96	101.25	1.34
**Asian or Pacific Islander**	2007	92.08	89.61	94.54	1.26
**Asian or Pacific Islander**	2008	88.37	86.04	90.71	1.19
**Asian or Pacific Islander**	2009	83.64	81.43	85.85	1.13
**Asian or Pacific Islander**	2010	83.68	81.52	85.84	1.1
**Asian or Pacific Islander**	2011	79.89	77.86	81.91	1.03
**Asian or Pacific Islander**	2012	77.07	75.16	78.98	0.98
**Asian or Pacific Islander**	2013	74.34	72.53	76.15	0.92
**Asian or Pacific Islander**	2014	71.35	69.65	73.06	0.87

**Asian or Pacific Islander**	2015	73.06	71.39	74.74	0.85
**Asian or Pacific Islander**	2016	74.64	72.99	76.28	0.84
**Asian or Pacific Islander**	2017	73.69	72.11	75.27	0.81
**Asian or Pacific Islander**	2018	73.13	71.59	74.67	0.78
**Asian or Pacific Islander**	2019	73.84	72.34	75.34	0.77
**Asian or Pacific Islander**	2020	84.02	82.46	85.58	0.8
**Asian or Pacific Islander**	2021	86	84.41	87.62	0.82
**Asian or Pacific Islander**	2022	79	77.54	80.49	0.75
**Asian or Pacific Islander**	2023	73.62	72.22	75.03	0.71
**Asian or Pacific Islander**	2024	72.02	70.7	73.36	0.68
**Black or African American**	1999	227.09	224.61	229.57	1.26
**Black or African American**	2000	226.63	224.17	229.09	1.25
**Black or African American**	2001	220.57	218.16	222.98	1.23
**Black or African American**	2002	214.71	212.34	217.08	1.21
**Black or African American**	2003	208.09	205.78	210.4	1.18

**Black or African American**	2004	197.13	194.9	199.36	1.14
**Black or African American**	2005	183.88	181.75	186	1.08
**Black or African American**	2006	172.95	170.92	174.99	1.04
**Black or African American**	2007	166.98	165	168.95	1.01
**Black or African American**	2008	158.7	156.79	160.6	0.97
**Black or African American**	2009	146.93	145.13	148.74	0.92
**Black or African American**	2010	145.03	143.25	146.8	0.9
**Black or African American**	2011	140.5	138.79	142.22	0.88
**Black or African American**	2012	135.64	133.99	137.3	0.85
**Black or African American**	2013	134.46	132.84	136.09	0.83
**Black or African American**	2014	133.36	131.77	134.95	0.81
**Black or African American**	2015	136.09	134.51	137.68	0.81
**Black or African American**	2016	134.76	133.21	136.31	0.79
**Black or African American**	2017	137.48	135.94	139.01	0.79
**Black or African American**	2018	136.28	134.77	137.78	0.77

**Black or African American**	2019	136.98	135.5	138.47	0.76
**Black or African American**	2020	163.68	162.07	165.28	0.82
**Black or African American**	2021	166.4	164.76	168.06	0.84
**Black or African American**	2022	160.18	158.6	161.77	0.81
**Black or African American**	2023	151.44	149.91	152.98	0.78
**Black or African American**	2024	148.37	146.89	149.85	0.75
**White**	1999	154	153.37	154.62	0.32
**White**	2000	151.66	151.04	152.28	0.32
**White**	2001	145.07	144.47	145.67	0.31
**White**	2002	142.54	141.95	143.14	0.3
**White**	2003	135.41	134.84	135.99	0.29
**White**	2004	127.26	126.7	127.81	0.28
**White**	2005	119.37	118.84	119.91	0.27
**White**	2006	111.49	110.98	112.01	0.26
**White**	2007	107.18	106.68	107.68	0.25
**White**	2008	103.8	103.31	104.29	0.25
**White**	2009	98.34	97.87	98.82	0.24
**White**	2010	97.52	97.05	97.99	0.24
**White**	2011	95.48	95.02	95.93	0.23
**White**	2012	93.54	93.09	93.99	0.23
**White**	2013	91.61	91.17	92.05	0.23
**White**	2014	91.75	91.31	92.19	0.22
**White**	2015	93.94	93.5	94.39	0.23

**White**	2016	93.54	93.1	93.98	0.22
**White**	2017	94.63	94.19	95.07	0.22
**White**	2018	93.96	93.53	94.4	0.22
**White**	2019	94.39	93.96	94.82	0.22
**White**	2020	104.28	103.83	104.73	0.23
**White**	2021	112.38	111.89	112.86	0.25
**White**	2022	107.74	107.28	108.2	0.23
**White**	2023	104.82	104.37	105.28	0.23
**White**	2024	104.01	103.56	104.46	0.23
**Hispanic**	1999	127.32	124.74	129.89	1.31
**Hispanic**	2000	124.08	121.6	126.55	1.26
**Hispanic**	2001	120.45	118.08	122.81	1.21
**Hispanic**	2002	117.43	115.14	119.71	1.16
**Hispanic**	2003	114.15	111.96	116.34	1.12
**Hispanic**	2004	107.94	105.86	110.01	1.06
**Hispanic**	2005	103.51	101.54	105.47	1
**Hispanic**	2006	97.58	95.72	99.44	0.95
**Hispanic**	2007	92.91	91.15	94.68	0.9
**Hispanic**	2008	87.99	86.32	89.66	0.85
**Hispanic**	2009	83.8	82.22	85.38	0.81
**Hispanic**	2010	84.59	83.03	86.14	0.79
**Hispanic**	2011	80.24	78.79	81.7	0.74
**Hispanic**	2012	78.74	77.34	80.13	0.71
**Hispanic**	2013	77.64	76.29	78.99	0.69
**Hispanic**	2014	77.89	76.58	79.2	0.67
**Hispanic**	2015	81.21	79.92	82.51	0.66
**Hispanic**	2016	80.9	79.64	82.16	0.64
**Hispanic**	2017	79.85	78.63	81.07	0.62

**Hispanic**	2018	80.13	78.94	81.33	0.61
**Hispanic**	2019	81.75	80.56	82.93	0.6
**Hispanic**	2020	96.24	94.99	97.49	0.64
**Hispanic**	2021	97.26	96	98.53	0.64
**Hispanic**	2022	93.08	91.88	94.29	0.61
**Hispanic**	2023	88.12	86.97	89.29	0.59
**Hispanic**	2024	85.46	84.37	86.57	0.56

Supplemental Table S4. Cerebrovascular Disease-Related Mortality Rates Stratified by Age

Ten-Year Age Groups	Year	Crude Rate	Crude Rate Lower 95% Confidence Interval	Crude Rate Upper 95% Confidence Interval	Crude Rate Standard Error
**25-34 years**	1999	2.54	2.39	2.7	0.08
**25-34 years**	2000	2.49	2.33	2.64	0.08
**25-34 years**	2001	2.66	2.5	2.82	0.08
**25-34 years**	2002	2.52	2.36	2.68	0.08
**25-34 years**	2003	2.57	2.41	2.72	0.08
**25-34 years**	2004	2.52	2.36	2.68	0.08
**25-34 years**	2005	2.39	2.23	2.54	0.08
**25-34 years**	2006	2.34	2.19	2.49	0.08
**25-34 years**	2007	2.21	2.06	2.35	0.07
**25-34 years**	2008	2.29	2.14	2.44	0.08
**25-34 years**	2009	2.3	2.15	2.45	0.08
**25-34 years**	2010	2.22	2.07	2.36	0.07
**25-34 years**	2011	2.28	2.14	2.43	0.07
**25-34 years**	2012	2.32	2.18	2.47	0.07

**25-34 years**	2013	2.15	2.01	2.29	0.07
**25-34 years**	2014	2.33	2.19	2.47	0.07
**25-34 years**	2015	2.48	2.33	2.63	0.07
**25-34 years**	2016	2.58	2.43	2.73	0.08
**25-34 years**	2017	2.58	2.44	2.73	0.08
**25-34 years**	2018	2.66	2.51	2.81	0.08
**25-34 years**	2019	2.63	2.49	2.78	0.08
**25-34 years**	2020	2.86	2.71	3.02	0.08
**25-34 years**	2021	3.22	3.06	3.39	0.08
**25-34 years**	2022	3.01	2.85	3.17	0.08
**25-34 years**	2023	2.78	2.63	2.94	0.08
**25-34 years**	2024	2.54	2.4	2.69	0.07
**35-44 years**	1999	8.84	8.57	9.11	0.14
**35-44 years**	2000	8.72	8.45	8.99	0.14
**35-44 years**	2001	8.46	8.19	8.73	0.14
**35-44 years**	2002	8.5	8.23	8.77	0.14
**35-44 years**	2003	8.57	8.3	8.85	0.14
**35-44 years**	2004	8.23	7.96	8.5	0.14
**35-44 years**	2005	7.91	7.64	8.17	0.13
**35-44 years**	2006	7.84	7.58	8.1	0.13
**35-44 years**	2007	7.68	7.41	7.94	0.13
**35-44 years**	2008	7.2	6.94	7.45	0.13
**35-44 years**	2009	7.1	6.84	7.35	0.13
**35-44 years**	2010	6.93	6.68	7.19	0.13
**35-44 years**	2011	6.76	6.51	7.01	0.13
**35-44 years**	2012	6.62	6.37	6.88	0.13
**35-44 years**	2013	6.72	6.47	6.97	0.13
**35-44 years**	2014	6.96	6.7	7.22	0.13

**35-44 years**	2015	7.18	6.92	7.44	0.13
**35-44 years**	2016	7.55	7.28	7.82	0.14
**35-44 years**	2017	7.43	7.17	7.69	0.13
**35-44 years**	2018	7.16	6.91	7.42	0.13
**35-44 years**	2019	7.47	7.21	7.73	0.13
**35-44 years**	2020	8.66	8.38	8.94	0.14
**35-44 years**	2021	9.79	9.5	10.09	0.15
**35-44 years**	2022	9.23	8.94	9.51	0.15
**35-44 years**	2023	8.7	8.42	8.97	0.14
**35-44 years**	2024	8.6	8.33	8.87	0.14
**45-54 years**	1999	24.19	23.68	24.69	0.26
**45-54 years**	2000	24.77	24.26	25.27	0.26
**45-54 years**	2001	23.76	23.28	24.24	0.25
**45-54 years**	2002	23.72	23.24	24.2	0.24
**45-54 years**	2003	23.77	23.3	24.24	0.24
**45-54 years**	2004	23.49	23.03	23.96	0.24
**45-54 years**	2005	23.26	22.8	23.72	0.23
**45-54 years**	2006	22.76	22.32	23.21	0.23
**45-54 years**	2007	22.4	21.95	22.84	0.23
**45-54 years**	2008	21.55	21.12	21.98	0.22
**45-54 years**	2009	21.37	20.95	21.8	0.22
**45-54 years**	2010	20.46	20.04	20.87	0.21
**45-54 years**	2011	20.65	20.23	21.07	0.21
**45-54 years**	2012	20.25	19.83	20.67	0.21
**45-54 years**	2013	20.24	19.82	20.66	0.22
**45-54 years**	2014	20.01	19.59	20.43	0.21
**45-54 years**	2015	20.35	19.92	20.77	0.22
**45-54 years**	2016	20.69	20.26	21.12	0.22

**45-54 years**	2017	20.67	20.24	21.1	0.22
**45-54 years**	2018	20.94	20.5	21.38	0.22
**45-54 years**	2019	21.73	21.28	22.18	0.23
**45-54 years**	2020	25.24	24.75	25.73	0.25
**45-54 years**	2021	26.87	26.37	27.37	0.26
**45-54 years**	2022	25.16	24.67	25.65	0.25
**45-54 years**	2023	23.76	23.29	24.24	0.24
**45-54 years**	2024	23.11	22.64	23.57	0.24
**55-64 years**	1999	71.32	70.24	72.39	0.55
**55-64 years**	2000	71.08	70.02	72.14	0.54
**55-64 years**	2001	67.13	66.11	68.14	0.52
**55-64 years**	2002	64.52	63.56	65.48	0.49
**55-64 years**	2003	62.43	61.51	63.36	0.47
**55-64 years**	2004	59.45	58.57	60.33	0.45
**55-64 years**	2005	58.06	57.21	58.91	0.44
**55-64 years**	2006	56.16	55.34	56.98	0.42
**55-64 years**	2007	54.1	53.31	54.89	0.4
**55-64 years**	2008	51.62	50.86	52.38	0.39
**55-64 years**	2009	50.73	49.99	51.47	0.38
**55-64 years**	2010	50.33	49.6	51.06	0.37
**55-64 years**	2011	50.35	49.63	51.06	0.36
**55-64 years**	2012	49.9	49.19	50.6	0.36
**55-64 years**	2013	49.82	49.13	50.52	0.36
**55-64 years**	2014	51.16	50.46	51.86	0.36
**55-64 years**	2015	51.78	51.09	52.48	0.36
**55-64 years**	2016	51.81	51.12	52.5	0.35
**55-64 years**	2017	53.01	52.31	53.7	0.36
**55-64 years**	2018	54.05	53.35	54.75	0.36

**55-64 years**	2019	55.53	54.82	56.24	0.36
**55-64 years**	2020	65.14	64.37	65.91	0.39
**55-64 years**	2021	68.38	67.6	69.17	0.4
**55-64 years**	2022	65.46	64.68	66.23	0.39
**55-64 years**	2023	61.59	60.84	62.34	0.38
**55-64 years**	2024	60.28	59.54	61.03	0.38
**65-74 years**	1999	239.6	237.36	241.83	1.14
**65-74 years**	2000	233.52	231.31	235.73	1.13
**65-74 years**	2001	224.15	221.99	226.31	1.1
**65-74 years**	2002	216.71	214.58	218.84	1.09
**65-74 years**	2003	203.2	201.15	205.25	1.05
**65-74 years**	2004	192.41	190.42	194.4	1.02
**65-74 years**	2005	180.85	178.93	182.77	0.98
**65-74 years**	2006	170.32	168.47	172.16	0.94
**65-74 years**	2007	162.43	160.65	164.21	0.91
**65-74 years**	2008	154.2	152.5	155.9	0.87
**65-74 years**	2009	147.19	145.56	148.83	0.83
**65-74 years**	2010	144.26	142.66	145.86	0.82
**65-74 years**	2011	140.75	139.2	142.31	0.79
**65-74 years**	2012	135.37	133.9	136.84	0.75
**65-74 years**	2013	133.61	132.18	135.03	0.73
**65-74 years**	2014	131.88	130.49	133.27	0.71
**65-74 years**	2015	134.88	133.5	136.25	0.7
**65-74 years**	2016	136.29	134.94	137.64	0.69
**65-74 years**	2017	137.25	135.92	138.58	0.68
**65-74 years**	2018	138.08	136.76	139.4	0.67
**65-74 years**	2019	139.1	137.8	140.41	0.66
**65-74 years**	2020	160.94	159.56	162.32	0.7

**65-74 years**	2021	166.47	165.09	167.85	0.7
**65-74 years**	2022	164.16	162.79	165.52	0.7
**65-74 years**	2023	152.32	151.02	153.62	0.66
**65-74 years**	2024	151.27	149.99	152.55	0.65
**75-84 years**	1999	808.48	803.44	813.52	2.57
**75-84 years**	2000	792.99	788.03	797.95	2.53
**75-84 years**	2001	759.64	754.82	764.45	2.46
**75-84 years**	2002	739.69	734.97	744.4	2.41
**75-84 years**	2003	703.89	699.31	708.47	2.34
**75-84 years**	2004	664.89	660.45	669.32	2.26
**75-84 years**	2005	617.18	612.92	621.44	2.17
**75-84 years**	2006	577.62	573.5	581.73	2.1
**75-84 years**	2007	550.41	546.39	554.43	2.05
**75-84 years**	2008	534.58	530.62	538.54	2.02
**75-84 years**	2009	502.65	498.8	506.5	1.96
**75-84 years**	2010	494.69	490.88	498.5	1.95
**75-84 years**	2011	487.09	483.32	490.86	1.92
**75-84 years**	2012	469.44	465.75	473.13	1.88
**75-84 years**	2013	460.91	457.28	464.54	1.85
**75-84 years**	2014	452.36	448.8	455.92	1.82
**75-84 years**	2015	460.2	456.64	463.77	1.82
**75-84 years**	2016	450.25	446.77	453.74	1.78
**75-84 years**	2017	448.87	445.44	452.29	1.75
**75-84 years**	2018	440.06	436.75	443.38	1.69
**75-84 years**	2019	438.8	435.55	442.04	1.66
**75-84 years**	2020	489.43	486.05	492.81	1.72
**75-84 years**	2021	506	502.53	509.46	1.77
**75-84 years**	2022	488.26	484.98	491.53	1.67

**75-84 years**	2023	457.52	454.43	460.61	1.58
**75-84 years**	2024	454.79	451.78	457.8	1.54
**85+ years**	1999	2582.1	2566.65	2597.56	7.88
**85+ years**	2000	2548.97	2533.78	2564.17	7.75
**85+ years**	2001	2452.5	2437.72	2467.28	7.54
**85+ years**	2002	2432.63	2418.01	2447.26	7.46
**85+ years**	2003	2317.13	2303.01	2331.25	7.2
**85+ years**	2004	2143.02	2129.56	2156.47	6.87
**85+ years**	2005	2007.82	1995	2020.64	6.54
**85+ years**	2006	1849.99	1837.9	1862.07	6.17
**85+ years**	2007	1786.95	1775.28	1798.62	5.95
**85+ years**	2008	1731.6	1720.28	1742.91	5.77
**85+ years**	2009	1612.93	1602.19	1623.68	5.48
**85+ years**	2010	1624.14	1613.48	1634.8	5.44
**85+ years**	2011	1556.95	1546.74	1567.16	5.21
**85+ years**	2012	1545.9	1535.85	1555.94	5.12
**85+ years**	2013	1498.46	1488.7	1508.23	4.98
**85+ years**	2014	1515.94	1506.22	1525.67	4.96
**85+ years**	2015	1569.34	1559.55	1579.13	5
**85+ years**	2016	1559.76	1550.07	1569.45	4.94
**85+ years**	2017	1596.79	1587.05	1606.52	4.97
**85+ years**	2018	1580.46	1570.82	1590.09	4.91
**85+ years**	2019	1583.67	1574.08	1593.27	4.9
**85+ years**	2020	1746.72	1736.68	1756.76	5.12
**85+ years**	2021	1882.1	1871.11	1893.1	5.61
**85+ years**	2022	1772.84	1762.59	1783.09	5.23
**85+ years**	2023	1791.84	1781.3	1802.38	5.38
**85+ years**	2024	1764.65	1754.39	1774.92	5.24

Supplemental Table S5. Cerebrovascular Disease-Related Mortality Rates Stratified by Region

Census Region	Year	Age Adjusted Rate	Age Adjusted Rate Lower 95% Confidence Interval	Age Adjusted Rate Upper 95% Confidence Interval	Age Adjusted Rate Standard Error
**Northeast**	1999	133.08	131.91	134.25	0.59
**Northeast**	2000	132.34	131.19	133.5	0.59
**Northeast**	2001	126.37	125.25	127.49	0.57
**Northeast**	2002	122.88	121.79	123.98	0.56
**Northeast**	2003	116.6	115.53	117.66	0.54
**Northeast**	2004	110.06	109.03	111.09	0.53
**Northeast**	2005	102.53	101.54	103.52	0.5
**Northeast**	2006	96.25	95.29	97.2	0.49
**Northeast**	2007	92.81	91.88	93.74	0.47
**Northeast**	2008	88.92	88.01	89.82	0.46
**Northeast**	2009	84.96	84.08	85.84	0.45
**Northeast**	2010	84.73	83.85	85.6	0.45
**Northeast**	2011	84.09	83.22	84.95	0.44
**Northeast**	2012	81.78	80.94	82.63	0.43
**Northeast**	2013	80.5	79.67	81.34	0.43
**Northeast**	2014	79.63	78.8	80.46	0.42
**Northeast**	2015	80.57	79.75	81.4	0.42
**Northeast**	2016	78.31	77.5	79.12	0.41
**Northeast**	2017	77.66	76.86	78.45	0.41
**Northeast**	2018	76.66	75.87	77.44	0.4
**Northeast**	2019	76.2	75.43	76.98	0.4
**Northeast**	2020	86.11	85.28	86.93	0.42

**Northeast**	2021	85.45	84.62	86.28	0.42
**Northeast**	2022	82.04	81.25	82.84	0.41
**Northeast**	2023	78.16	77.38	78.94	0.4
**Northeast**	2024	76.64	75.89	77.41	0.39
**Midwest**	1999	165.97	164.75	167.2	0.62
**Midwest**	2000	160.43	159.23	161.63	0.61
**Midwest**	2001	154.15	152.98	155.32	0.6
**Midwest**	2002	151.39	150.24	152.54	0.59
**Midwest**	2003	142.86	141.75	143.97	0.57
**Midwest**	2004	135.02	133.94	136.09	0.55
**Midwest**	2005	128.37	127.33	129.41	0.53
**Midwest**	2006	119.98	118.98	120.98	0.51
**Midwest**	2007	115.19	114.22	116.16	0.5
**Midwest**	2008	113.13	112.17	114.09	0.49
**Midwest**	2009	105.35	104.43	106.27	0.47
**Midwest**	2010	104.36	103.45	105.27	0.46
**Midwest**	2011	102.71	101.82	103.61	0.46
**Midwest**	2012	99.51	98.64	100.38	0.45
**Midwest**	2013	97.52	96.66	98.38	0.44
**Midwest**	2014	98.33	97.47	99.19	0.44
**Midwest**	2015	98.21	97.36	99.06	0.43
**Midwest**	2016	97.56	96.71	98.4	0.43
**Midwest**	2017	99.65	98.8	100.49	0.43
**Midwest**	2018	98.78	97.94	99.61	0.42
**Midwest**	2019	99.85	99.02	100.68	0.42
**Midwest**	2020	114.65	113.76	115.53	0.45
**Midwest**	2021	117.89	116.98	118.82	0.47
**Midwest**	2022	112.53	111.65	113.41	0.45

**Midwest**	2023	110.98	110.11	111.86	0.45
**Midwest**	2024	111.93	111.07	112.8	0.44
**South**	1999	170.16	169.12	171.19	0.53
**South**	2000	169.98	168.95	171.01	0.52
**South**	2001	162.93	161.93	163.92	0.51
**South**	2002	159.93	158.94	160.91	0.5
**South**	2003	152.77	151.81	153.72	0.49
**South**	2004	142.93	142.02	143.85	0.47
**South**	2005	135.22	134.34	136.1	0.45
**South**	2006	126.14	125.31	126.98	0.43
**South**	2007	121.73	120.92	122.54	0.42
**South**	2008	116.68	115.89	117.47	0.4
**South**	2009	110.87	110.11	111.63	0.39
**South**	2010	110.02	109.27	110.77	0.38
**South**	2011	105.86	105.14	106.59	0.37
**South**	2012	104.3	103.59	105.01	0.36
**South**	2013	101.74	101.05	102.43	0.35
**South**	2014	102.16	101.47	102.84	0.35
**South**	2015	106.19	105.5	106.88	0.35
**South**	2016	105.3	104.62	105.97	0.34
**South**	2017	106.27	105.6	106.94	0.34
**South**	2018	106.82	106.16	107.49	0.34
**South**	2019	107.29	106.63	107.95	0.33
**South**	2020	119.8	119.12	120.49	0.35
**South**	2021	130.04	129.31	130.77	0.37
**South**	2022	124.53	123.84	125.23	0.35
**South**	2023	119.86	119.19	120.54	0.35
**South**	2024	118.06	117.4	118.72	0.34

**West**	1999	163.2	161.85	164.55	0.69
**West**	2000	158.61	157.29	159.93	0.67
**West**	2001	152.92	151.64	154.2	0.65
**West**	2002	150.09	148.84	151.35	0.64
**West**	2003	144.98	143.77	146.2	0.62
**West**	2004	135.36	134.2	136.53	0.59
**West**	2005	124.19	123.09	125.29	0.56
**West**	2006	116.57	115.52	117.62	0.54
**West**	2007	110.33	109.32	111.34	0.51
**West**	2008	106.26	105.28	107.24	0.5
**West**	2009	99.89	98.96	100.83	0.48
**West**	2010	98.36	97.44	99.28	0.47
**West**	2011	95.87	94.98	96.76	0.46
**West**	2012	93.63	92.76	94.5	0.44
**West**	2013	91.97	91.12	92.82	0.43
**West**	2014	90.7	89.87	91.53	0.42
**West**	2015	94.01	93.17	94.84	0.43
**West**	2016	95.27	94.44	96.1	0.42
**West**	2017	96.94	96.12	97.77	0.42
**West**	2018	94	93.2	94.81	0.41
**West**	2019	94.66	93.87	95.46	0.41
**West**	2020	104.77	103.94	105.59	0.42
**West**	2021	112.9	112.03	113.79	0.45
**West**	2022	107.29	106.46	108.13	0.42
**West**	2023	102.36	101.55	103.17	0.41
**West**	2024	99.67	98.89	100.46	0.4

Supplemental Table S6. Cerebrovascular Disease-Related Mortality Rates Stratified by State

State	Population (1999)	AAMR (1999)	Population (2024)	AAMR (2024)	Combined AAMR from 1999 to 2024
**Alabama**	2868102	183.55	3534667	113.69	144.98
**Alaska**	376602	174.66	497552	105.28	135.17
**Arizona**	3192500	124.15	5266546	80.21	96.79
**Arkansas**	1709833	183.55	2097371	128.71	153.34
**California**	21033993	172.32	27361510	97.53	130.04
**Colorado**	2726699	135.26	4193325	109.75	119.80
**Connecticut**	2283090	131.98	2599722	73.19	100.68
**Delaware**	509177	163.59	748225	149.3	155.09
**District of Columbia**	386935	176.65	502330	106.62	137.09
**Florida**	10848678	126.29	16971145	115.32	119.60
**Georgia**	5098026	171.55	7576425	108.64	133.94
**Hawaii**	796535	168.1	1035937	92.59	125.41
**Idaho**	771756	166.54	1334105	100.52	124.72
**Illinois**	7926145	166.35	8837200	103.08	133.00
**Indiana**	3868825	175	4665592	118.56	144.15
**Iowa**	1890043	152.49	2183634	91.79	119.95
**Kansas**	1694402	146.05	1971456	98.67	120.57
**Kentucky**	2621057	181.05	3144460	117.88	146.60
**Louisiana**	2762515	176.79	3108310	129.1	151.54
**Maine**	860710	161.86	1045097	91.19	123.11
**Maryland**	3463880	183.48	4356279	132.83	155.27
**Massachusetts**	4254699	126.98	5070037	70.5	96.27
**Michigan**	6377862	166.88	7093655	123.62	144.10
**Minnesota**	3130423	151.68	3976277	118.48	133.10
**Mississippi**	1742845	188.23	1981752	142.97	164.15
**Missouri**	3605323	170.93	4301494	104.72	134.91

**Montana**	580980	150.19	802007	88.79	114.58
**Nebraska**	1081337	144.65	1320122	105.05	122.88
**Nevada**	1270129	138.32	2317096	103.54	115.85
**New Hampshire**	812571	147.86	1039873	86.58	113.46
**New Jersey**	5618014	135.68	6651323	72.49	101.42
**New Mexico**	1120522	135.02	1478068	86.16	107.23
**New York**	12464635	111.06	14096625	67.13	87.75
**North Carolina**	5225424	202.55	7634344	122.04	154.75
**North Dakota**	408740	163.03	520582	91.5	122.96
**Ohio**	7389311	178.48	8250595	123.67	149.57
**Oklahoma**	2187951	173.41	2724621	131	149.89
**Oregon**	2227784	183.71	3079436	140.64	158.72
**Pennsylvania**	8247805	157.94	9263235	93.87	124.05
**Rhode Island**	690314	131.15	796209	75.3	101.24
**South Carolina**	2564228	205.03	3827007	122.73	155.75
**South Dakota**	469979	150.61	617499	102.55	123.32
**Tennessee**	3708799	195.52	4998156	126.7	156.01
**Texas**	12570920	177.11	20553433	107.39	133.85
**Utah**	1177613	146.52	2152060	86.03	107.42
**Vermont**	401296	150.61	472039	97.71	122.02
**Virginia**	4610994	172.08	6113034	109.56	136.44
**Washington**	3786793	172.89	5627968	112.29	136.66
**West Virginia**	1229266	186.18	1265312	121.9	153.58
**Wisconsin**	3451577	162.57	4155423	106.76	132.08
**Wyoming**	311132	151.6	404917	98.93	121.82
